# Third molar transplantation combined with an osteotome sinus lift – two case reports

**DOI:** 10.1002/ccr3.1042

**Published:** 2017-06-28

**Authors:** Katsuki Yamamoto, Yoshinaga Osamu, Kae Kakura, Kazuko Yamamoto, Hirofumi Kido

**Affiliations:** ^1^ Section of Oral Implantology Department of Oral Rehabilitation Fukuoka Dental College Fukuoka Japan

**Keywords:** Dental implant, maxillary sinus floor elevation, tooth transplantation

## Abstract

We report here on our application of the socket lift technique to create a transplant socket for the transplanted maxillary molar. These outcomes suggest that this technique is likely to be effective for tooth transplantation when the maxillary sinus bottom is close to the alveolar crest.

## Introduction

Tooth transplantation was first reported in the 1950s, showing the potential for prosthesis treatment for lost teeth 1, 2, 3, 4, 5, 6. Since then, many successful natural tooth transplantations have been described.

Patel et al. 7 reported a survival rate of 83% for teeth with complete root formation over approximately 14 years of follow‐up observation. Nagori et al. 8 also reported a survival rate of 86% for transplanted teeth, including maxillary and mandibular teeth, indicating the usefulness of tooth transplants. According to several research and clinical reports, tooth transplants are currently an established form of treatment for dental deficits.

The most common indication for dental implant treatment of the maxillary molars is narrowing of the distance between the floor of the maxillary sinus and the alveolar crest. When treating these kinds of cases, the dental implant is inserted while making concomitant use of the socket lift technique. The socket lift technique is a type of maxillary sinus floor augmentation used to enable insertion of the dental implant into the deficit and is already an established technique 9, 10.

Treatment involving maxillary sinus floor augmentation and dental implant insertion has been established for cases with restrictions caused by the maxillary sinus, and there have been many reports of favorable clinical outcomes. However, there are very few reports on the combination of transplantation of natural teeth and maxillary sinus floor augmentation. For this reason, we report here on our application of the socket lift technique to create a transplant socket for the transplanted maxillary molar. The transplant of a maxillary molar is commonly subject to anatomical restrictions caused by the maxillary sinus.

## Materials and Methods

### Case 1

**Figure 1:**
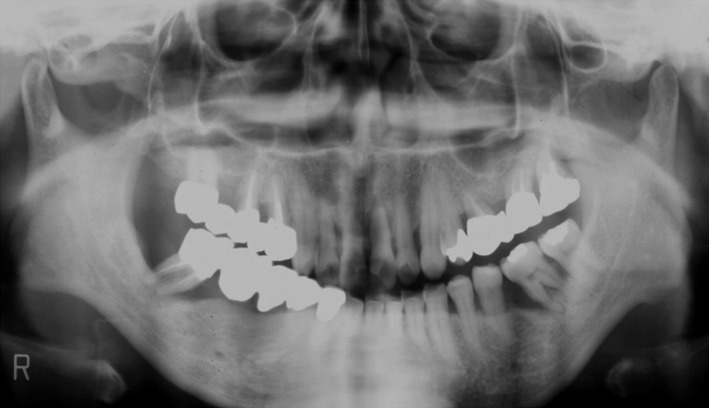
This patient was a 41 year old female. Her chief complaint was an occlusive pain in the upper left molar.

A 41‐year‐old female first visited our clinic for a feeling of strangeness in the left upper molar region in August 2000. The left mandibular third molar was transplanted to the left maxillary second molar part (Fig. 1).

**Figure 2:**
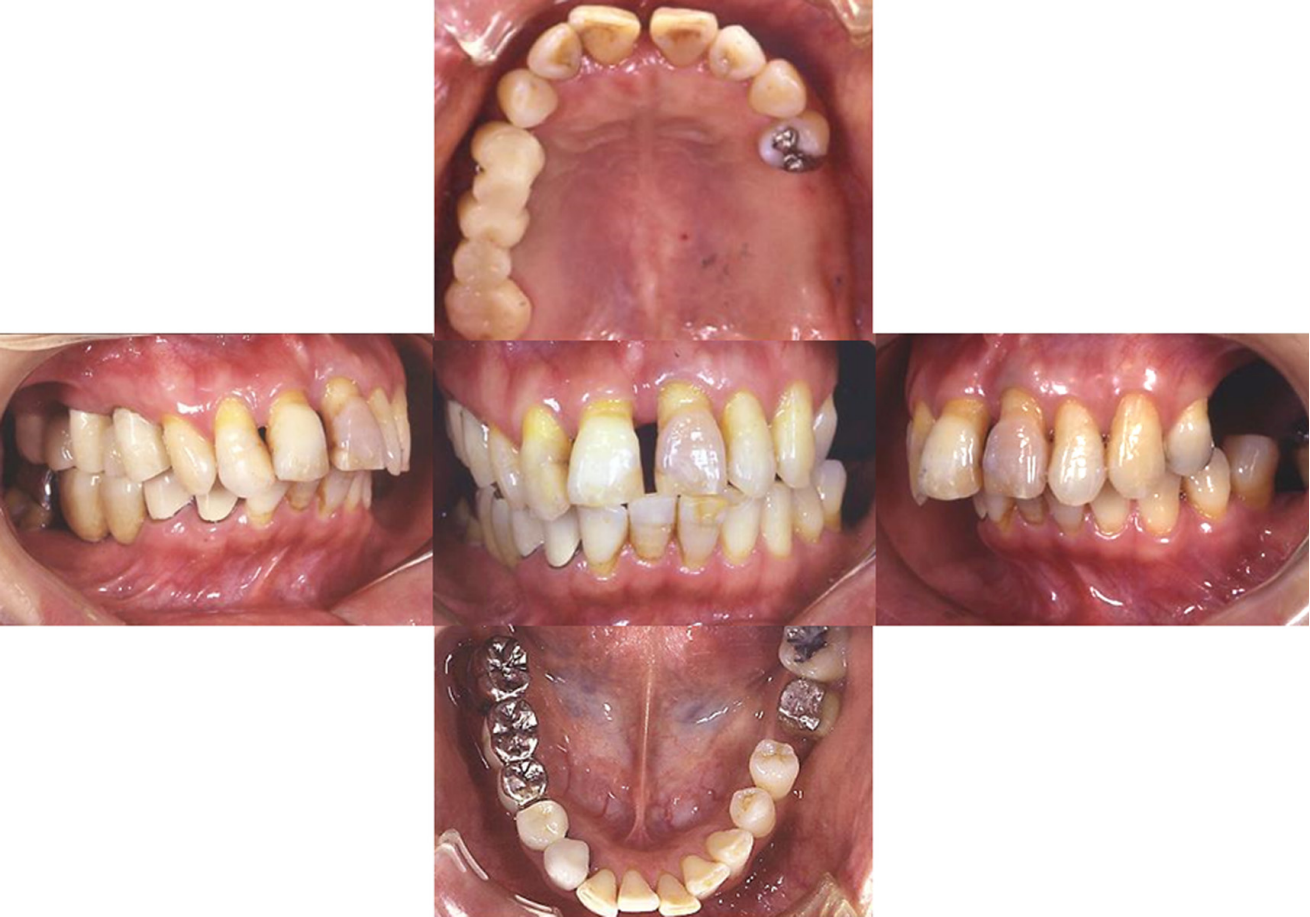
Tooth number 15, 16 and 31 was diagnosed to be extracted, while number 18 was to be preserved.

**Figure 3:**
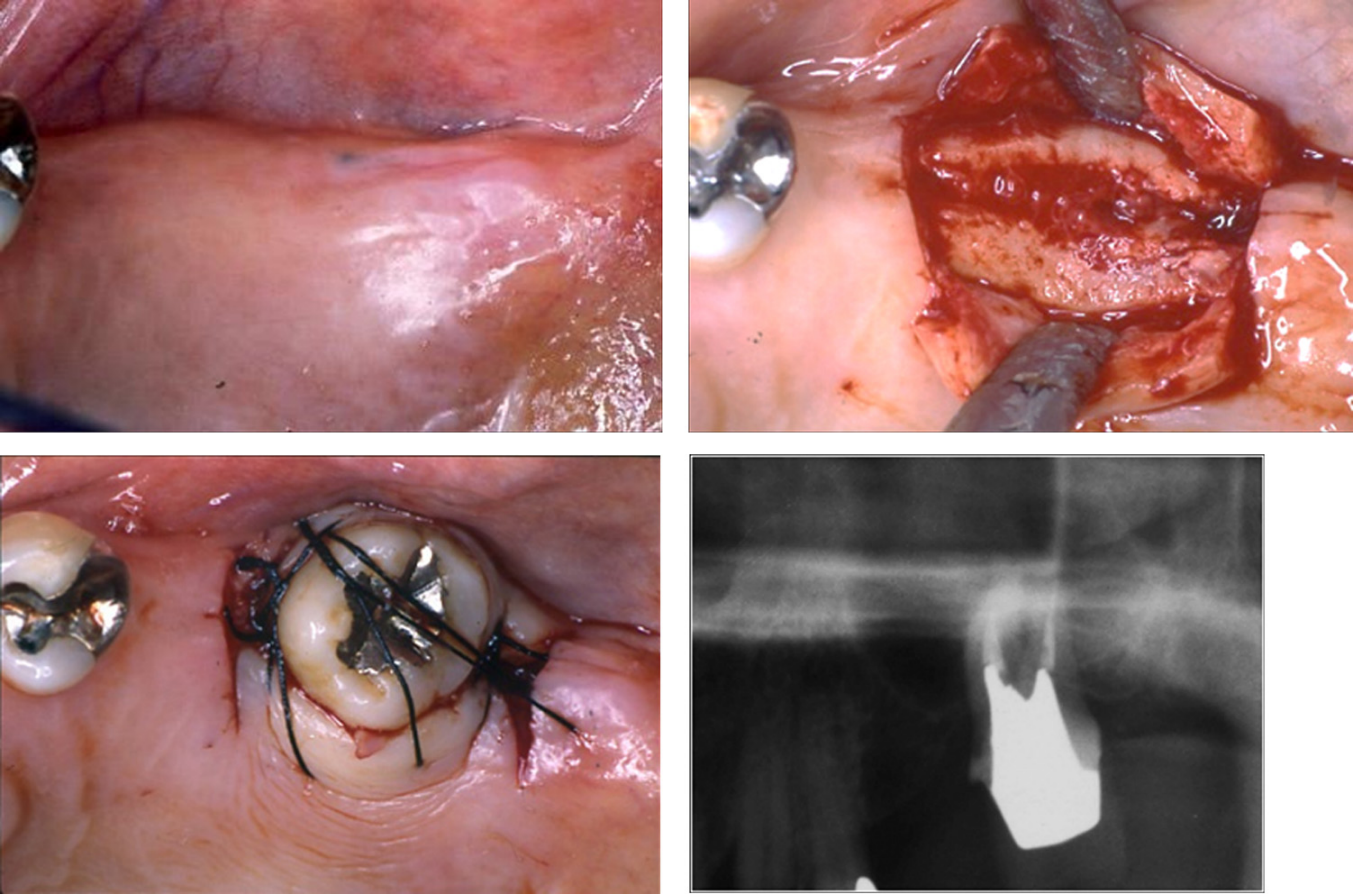
Tooth number 17 was transplanted to the position of tooth number15.

Due to severe periodontal disease, upper left #7 could not be preserved and we explained to the patient that they would require tooth extraction. We explained that they would be managed with dentures and oral implant treatments or tooth transplantation after extraction (Fig. 2). The patient requested tooth transplantation, so we performed a transplant of left maxillary second molar using lower left #8 as a donor tooth. During the transplant surgery, local infiltration anesthesia was administered to left maxillary second molar and left mandibular third molar, and then, left mandibular third molar was extracted with the utmost care to avoid injury to the periodontal ligament. Next, we performed drilling to create the transplant bed and created the space to insert a round bur and the implant. We then elevated the maxillary sinus floor using an osteotome. In order to conduct tooth transplantation, the shape of the tooth removed for transplantation and attachment condition of the periodontal membrane was measured using a probe. Based on the length and shape of the root of the transplanting tooth, a three‐dimensional transplant socket, which was slightly bigger than the root of the transplanting tooth, was created. Finally, the transplanting tooth was placed into the transplant socket, and fitting was adjusted by grinding the constricted part based on the pulling out resistance of the transplanting tooth. The left mandibular third molar was transplanted into the transplant socket and secured using sutures (Fig. 3). The patient was administered antibiotics and analgesics after surgery. The postoperative course was favorable.

**Figure 4:**
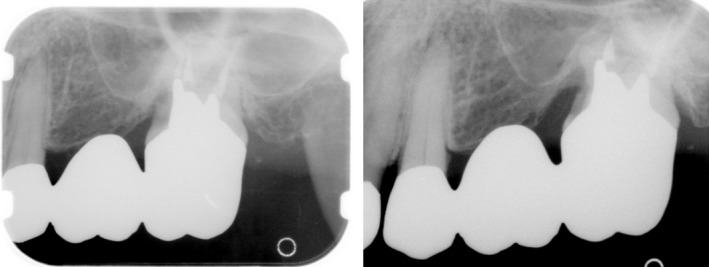
Dental X‐Rays at five year and 15 years post‐operative : No bone loss in the transplanted tooth and no other significant findings. No mobility, either.

About 3 weeks after transplantation, endodontic treatment was started for the transplanted tooth, and abutment construction was performed with a metal core after root canal obturation. Prosthesis treatment was performed for the defective left maxillary first molar, using the left maxillary second premolar and maxillary second molar as abutment teeth for bridging (Fig. 4).

### Case 2

**Figure 5:**
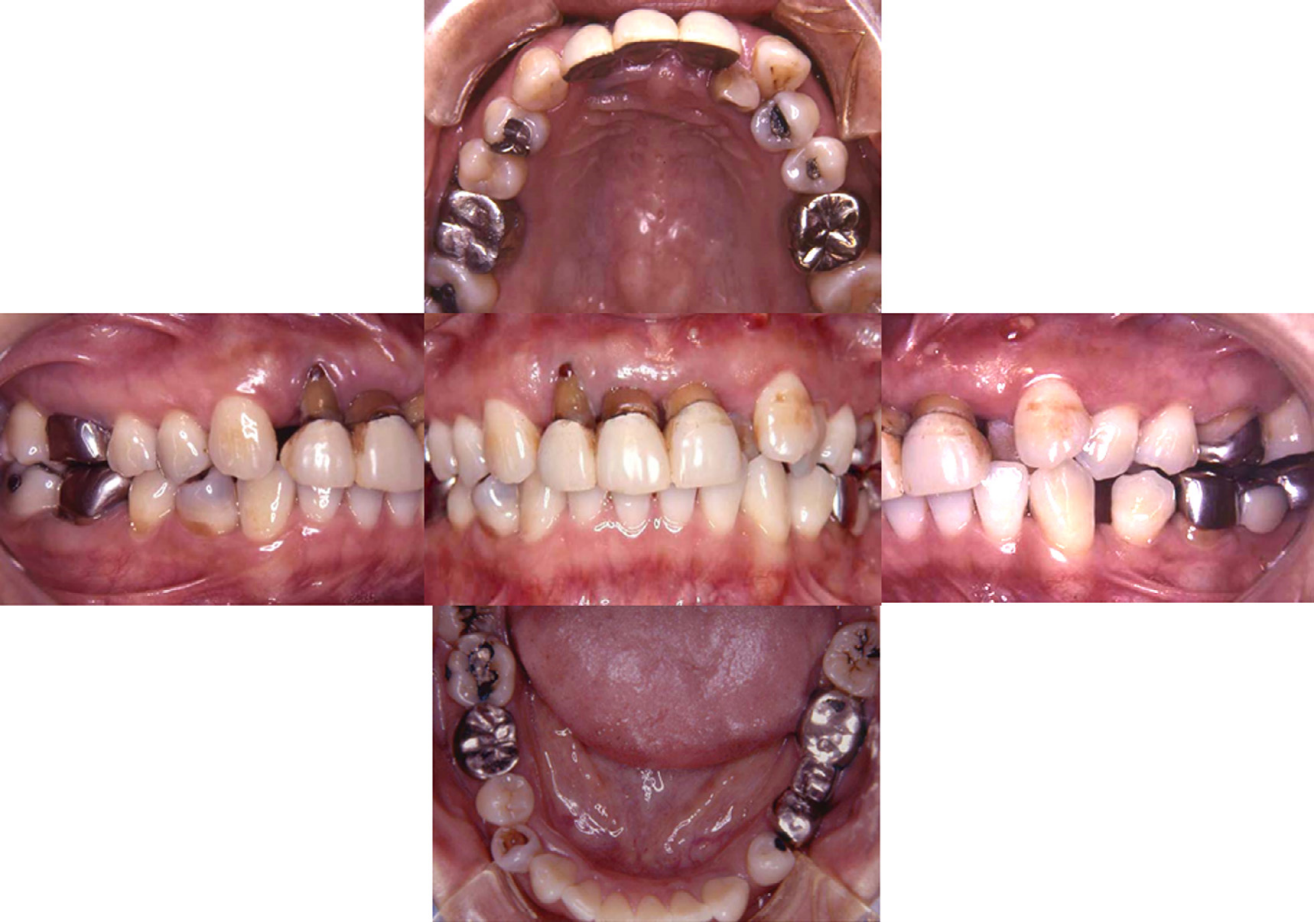
The patient was a 46 year old female, visited our clinic on June 2002.Her chief complaint was that she was in need of complete dental treatment.

**Figure 6:**
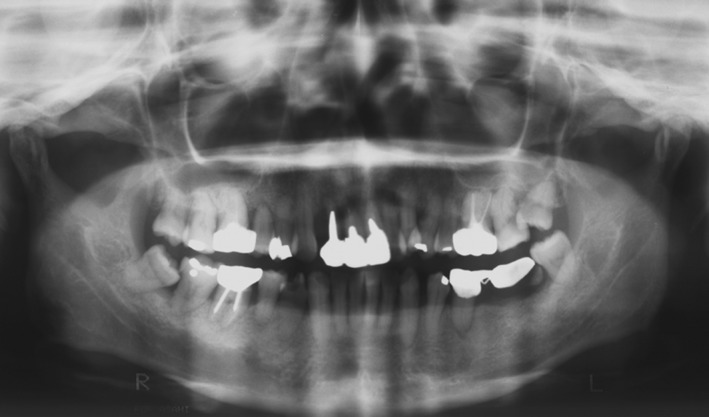
Panorama radiograph at the first visit.

A 46‐year‐old female first visited our clinic for cold‐water pain in the left upper back teeth in June 2002. The patient desired a treatment strategy with transplantation of natural tooth to the left maxillary second molar (Figs 5 and 6).

Digital X‐ray images were used to image the center of left maxillary second molar, where C3 dental caries were observed. Detailed investigations determined that removal of the softened dentin to treat the dental caries would extend up to the osseous margin. For this reason, we explained the treatment options for upper left maxillary second molar, including surgical treatment or elevation treatment for correction, tooth extraction and prosthetic management using dentures, tooth transplantation using natural teeth, oral implant therapy, or performing no treatment. We also explained the advantages and disadvantages of these methods of treatment. The patient requested tooth transplantation of upper maxillary second molar using natural teeth.

**Figure 7:**
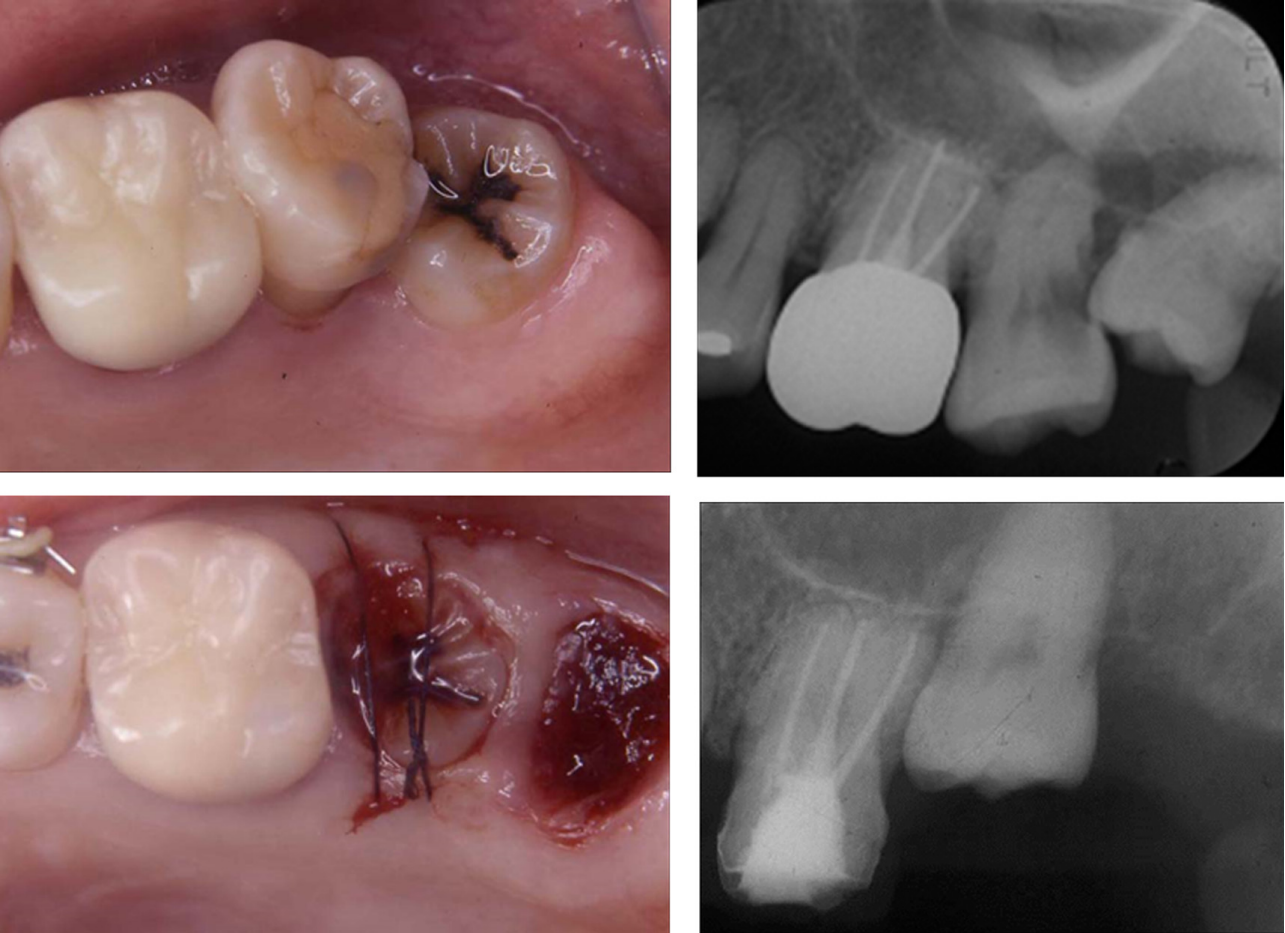
Tooth number 15 was extracted. The maxillary sinus was so low as be close to the apex portion of the tooth. For this reason, Tooth number 15 and 16 was extracted, and number 16 was transplanted into the cavity of number 15.

**Figure 8:**
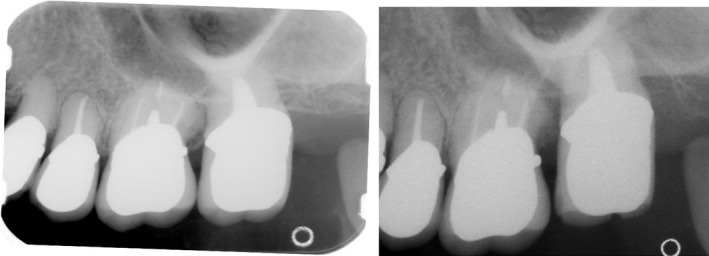
Dental X‐Rays at five year and 13 years post‐operative: No bone loss in the transplanted tooth and no other significant findings. No mobility, either.

Local infiltration anesthesia was performed for the left maxillary third molar and maxillary second molar to extract the left maxillary second molar. Curettage of a fossa for the extracted tooth was then immediately performed. Next, the third molar tooth was removed and drilling was performed using a round bur and a drill for formation of a fossa for the implant based on the width of the transplanted tooth root, so that the fossa would match the transplanted tooth. An apparatus for sinus lift (Osteotome) was inserted into the formed fossa and the maxillary sinus bottom was lifted up by tapping with a mallet. Then, the extracted left upper 8 was transplanted into the formed fossa. The transplanted tooth was fixed with suture, and the surgery was completed after confirming arrest of hemorrhage (Fig. 7). About 3 week after transplantation, root canal treatment for the transplanted tooth was started, and then, a porcelain fused metal crown was placed (Fig. 8).

## Results

We performed tooth transplantation concomitantly with socket lift as a therapeutic method for lost teeth in two cases.

**Figure 9:**
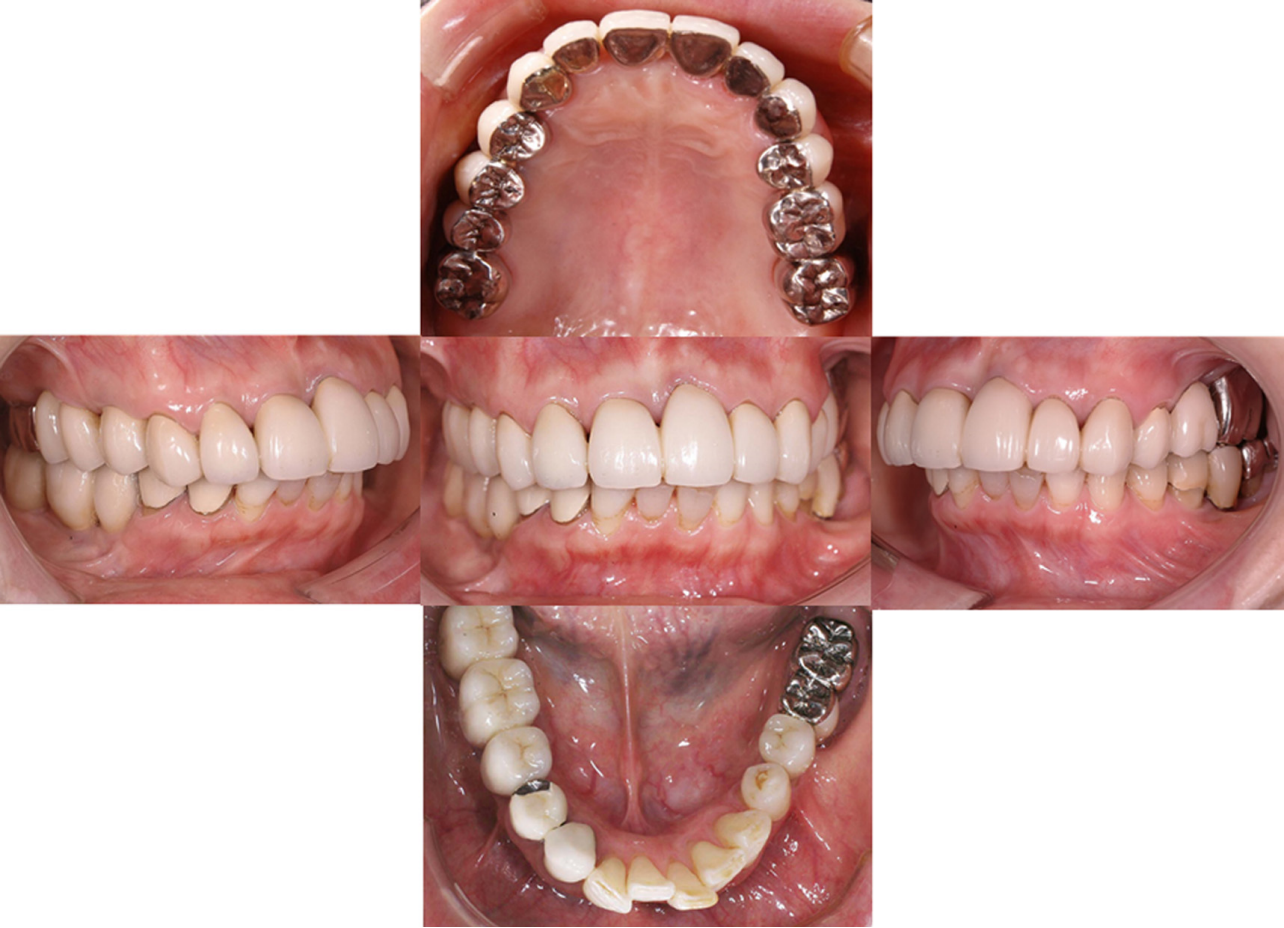
The final restoration : All of which were the Fixed partial dentures.

**Figure 10:**
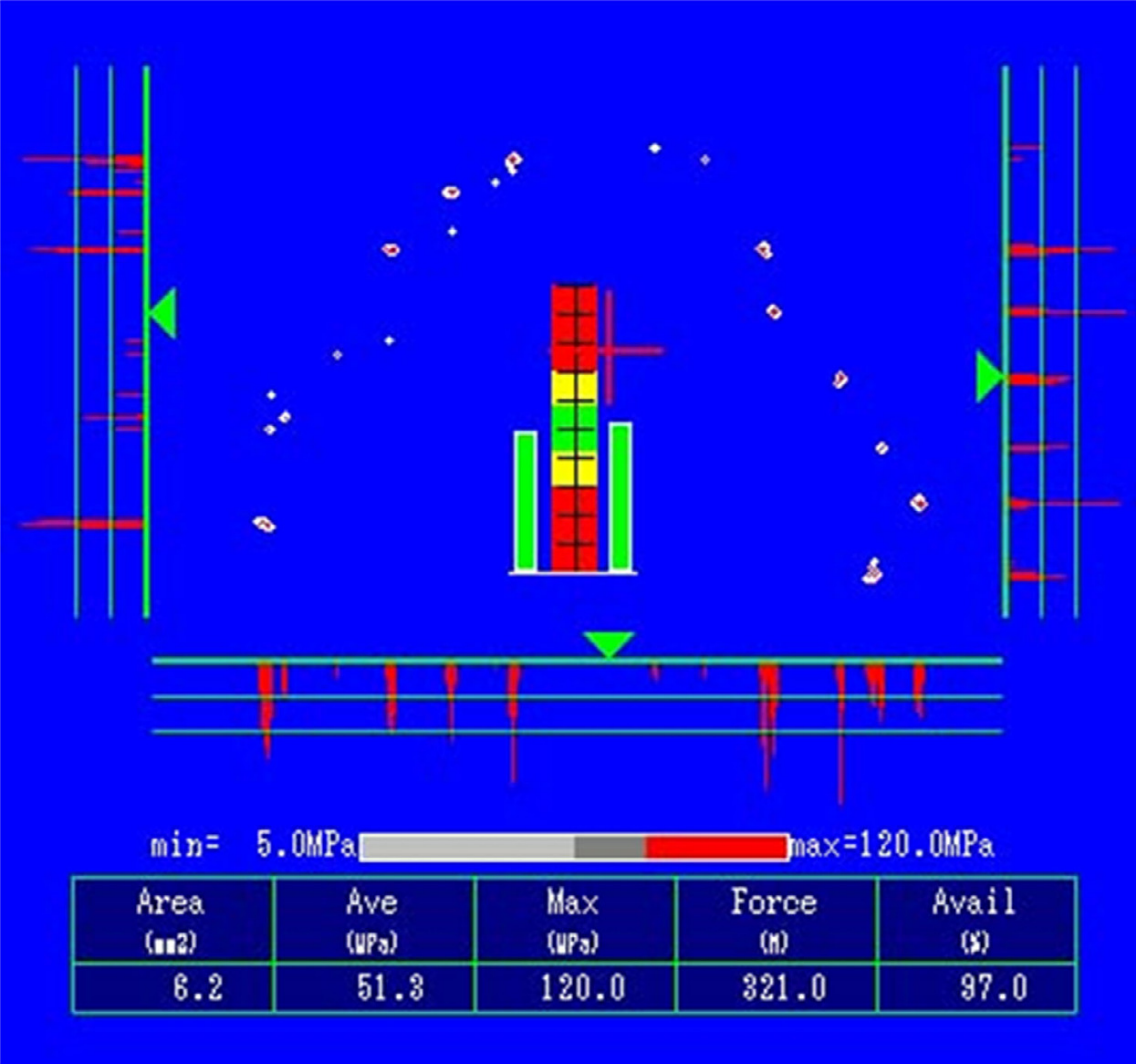
The occlusion was stable and occlusal force of 321N was recorded. The transplanted tooth was also in occlusion.

**Figure 11:**
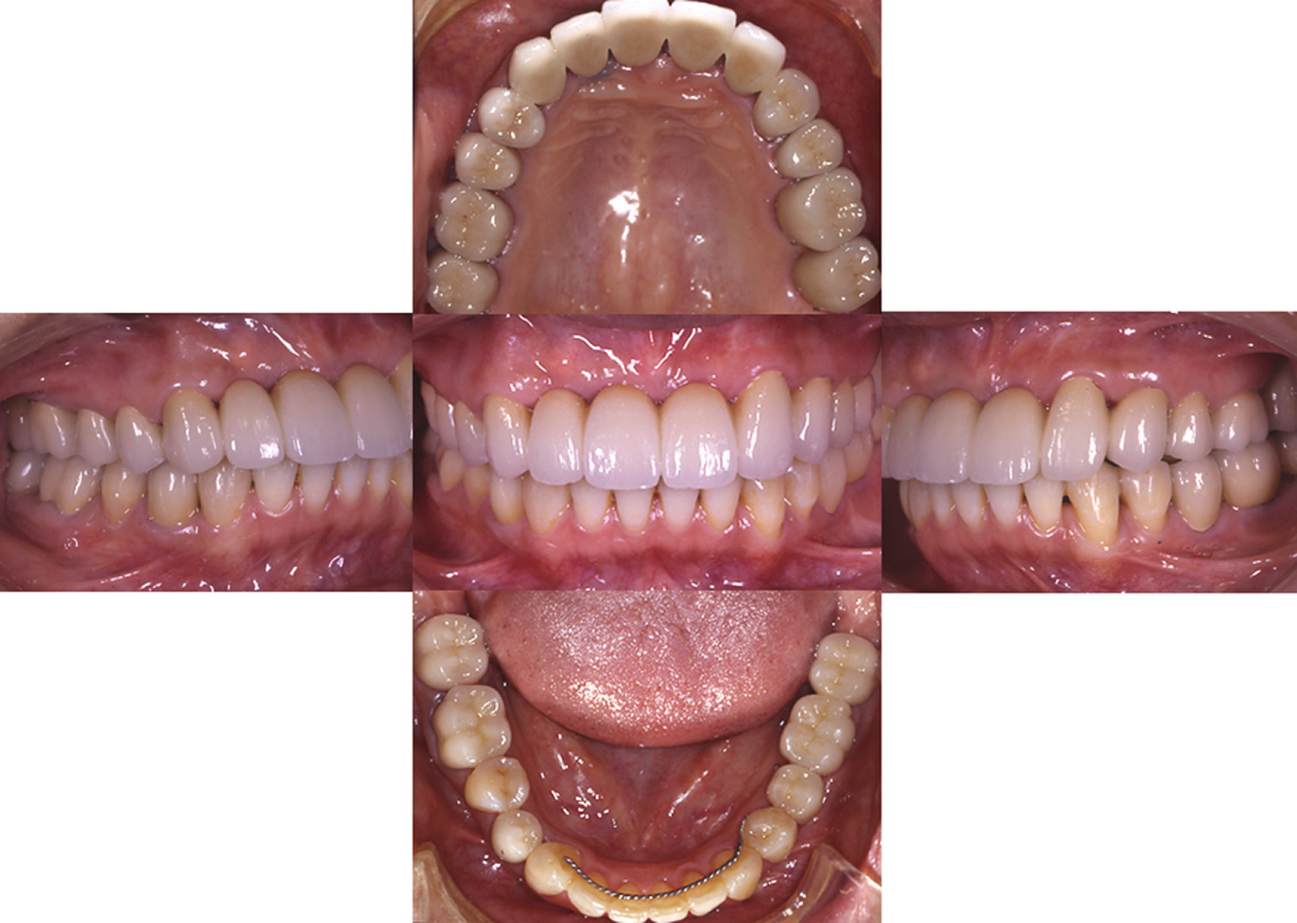
The final restoration : The teeth from number 5 to 12 were used as a bridge. The teeth from number 7 to 10 were “three incisors.” All the other natural teeth were restored with single crowns.

**Figure 12:**
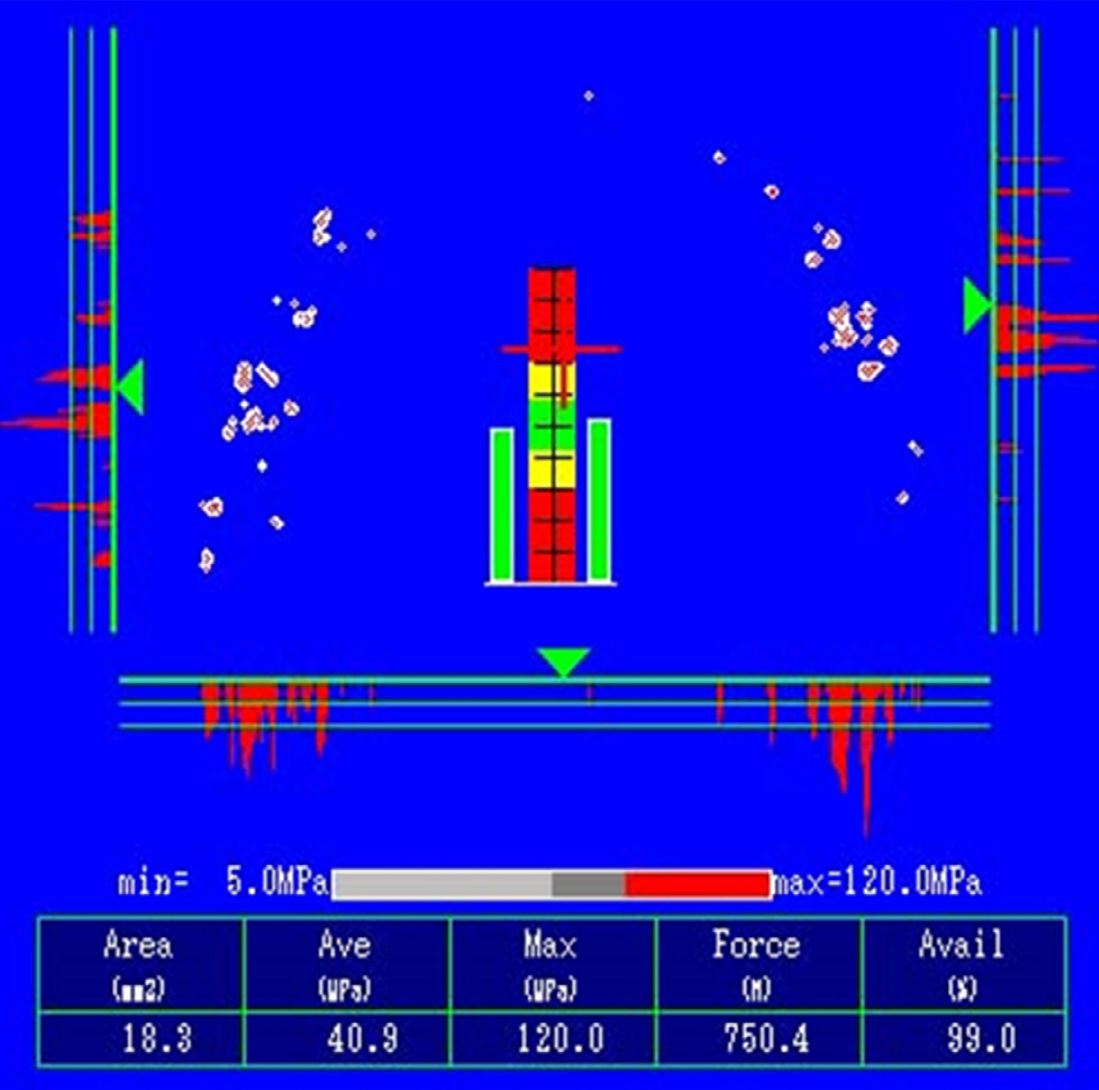
The patient's occlusal force and balance five years after the operation, measured by the Dental Prescale^®^.

Case 1: There have been no problems during follow‐up observation, and the patient has favorable articulation (Fig. 9). On the Dental Prescale^®^ at 5 years, the articulation balance was good, the biting force was 321 N, and favorable articulation of the transplanted tooth was maintained (Fig. 10).
Case 2: The teeth from number 5 to 12 were used as a bridge. The teeth from number 7 to 10 were “three incisors.” There have been no problems during follow‐up observation, and the patient has favorable articulation (Fig. 11). On the Dental Prescale^®^ (GC Co., Japan) at 5 years, the articulation balance was good, the biting force was 750 N, and favorable articulation of the transplanted tooth was maintained (Fig. 12).

## Discussion

We have presented two cases in which there was narrowing of the space between the maxillary sinus floor and the alveolar crest. We made concomitant use of the socket lift technique during tooth transplantation; the prognosis was favorable and we were able to perform long‐term follow‐up. In the first case, it has been 10 years since we transplanted upper left maxillary second molar using lower left #8 as the donor tooth. In the second case, approximately 10 years have passed since we performed a transplant of left maxillary second molar using left mandibular third molar as a donor tooth. The occlusal relationship obtained and the clinical course were both favorable.

Yan et al. 11 performed transplants using the third mandibular molars, reporting a survival rate of 94.3% after a mean period of 5.2 years of follow‐up. I‐Kai et al. 12 performed autologous transplantation involving the maxilla and mandible on 176 patients, reporting a survival rate of 82% after 5 years of follow‐up. Watanabe et al. 13 also reported a survival rate of 86.8% after 6 or more years of follow‐up of 38 cases of tooth transplantation. This was said to be due to the effects of endodontic treatment of the transplanted tooth, as well as avoiding injury to the periodontal ligament at the time of tooth extraction. Successful tooth transplantation requires careful attention to factors such as dental extraction without damage to the periodontal membrane, successive fixation of the transplanted tooth, close suture with soft tissues, and endodontic procedures. Survival of transplanted teeth has increased with the growing consensus on surgical methods and postoperative procedures.Nyman et al. 14 performed an experiment using extracted teeth. The periodontal ligament was removed from some of the teeth, and left intact on others, and tooth transplantation was performed. Results showed that no root resorption was observed when the teeth with periodontal ligaments were transplanted, even when they were in contact with either the alveolar bone or the mucosa. Andreasen et al. 15 conducted animal experiments and transplanted teeth after the periodontal ligament had been subjected to various artificial injuries in order to investigate the effects of periodontal ligament injury on its ability to regenerate. Results showed that the ability of the ligament to regenerate is maintained up to a certain extent of periodontal ligament injury, but that large injuries to the periodontal ligament will cause ankylosis. As suggested in the two cases described here, if a tooth with no serious damage to its periodontal membrane is transplanted to an elevated part of the maxillary sinus mucosa using the socket lift technique, the dental root may be protected by cells of the periodontal ligament, and regeneration of alveolar bone is likely. We reported the case of tooth transplantation, which applied the socket lift procedure in this paper. Good outcome of tooth transplantation requires sufficient healing of periodontal tissues after the transplantation. Lundgren et al. 16 performed maxillary sinus bottom elevation without bone transplantation for the insertion of dental implant in patients lacking a maxillary molar. As a result, they reported new bone formation around the implant inside the sinus. In addition, they suggested the importance of stimulating the bone surface for new bone formation by maxillary sinus bottom elevation.

Periodic follow‐up observation including maintenance is required in order to obtain a good prognosis. During the present study, we used a dental prescale as a method for objectively recording the occlusal relationship. The dental prescale uses specialized occlusal registration paper and allows dynamic changes in the occlusal contact area, mean pressure, maximum pressure, occlusal force and balance, and occlusion site to be recorded as numerical values 17. It is also used as an objective indicator for improving prosthetic treatment. In the present case, we were able to improve occlusion using the dental prescale, while also providing tightly controlled treatment follow‐up observation by evaluating the balance of the occlusal relationship numerically and visually.

## Conclusions

We reported two cases with favorable outcomes following use of socket lift to form a fossa for maxillary posterior tooth transplantation. The postoperative outcome of these cases has been favorable over 10 years. However, in tooth transplantation, it is not the case that everything about the healing process of the periodontium and mechanism of the root resorption, a fixed period was revealed sufficiently. Further studies are required to clarify the basic mechanisms, but these outcomes suggest that this technique is likely to be effective for tooth transplantation when the maxillary sinus bottom is close to the alveolar crest.

## Consent

Written informed consent was obtained from the patient for publication of this case report and any accompanying images.

## Authorship

KY: performed conception, interpretation, and critical revision of article. YO: performed conception and critical revision of article. KK, KY, and HK: contributed to approval of article and critical revision of article.

## Conflict of Interest

No conflict of interest.
